# Characterizing the Role of the miR156-SPL Network in Plant Development and Stress Response

**DOI:** 10.3390/plants9091206

**Published:** 2020-09-15

**Authors:** John Martin Jerome Jeyakumar, Asif Ali, Wen-Ming Wang, Muthu Thiruvengadam

**Affiliations:** 1 Rice Research Institute and Key Lab for Major Crop Diseases, Sichuan Agricultural University, Wenjiang, Chengdu 625014, China; jeromejeyakumarj@gmail.com (J.M.J.J.); asifalikalas@foxmail.com (A.A.); 2 State Key Laboratory of Crop Gene Exploration and Utilization in Southwest China, Institute of Rice Research, Sichuan Agricultural University, Wenjiang, Chengdu 625014, China; 3 Department of Applied Bioscience, College of Life and Environmental Sciences, Konkuk University, Seoul 05029, Korea; muthu@konkuk.ac.kr

**Keywords:** miR156, SPL, phase transition, secondary metabolites, abiotic stress

## Abstract

MicroRNA (miRNA) is a short, single-stranded, non-coding RNA found in eukaryotic cells that can regulate the expression of many genes at the post-transcriptional level. Among various plant miRNAs with diverse functions, miR156 plays a key role in biological processes, including developmental regulation, immune response, metabolic regulation, and abiotic stress. MiRNAs have become the regulatory center for plant growth and development. MicroRNA156 (miR156) is a highly conserved and emerging tool for the improvement of plant traits, including crop productivity and stress tolerance. Fine-tuning of squamosa promoter biding-like (SPL) gene expression might be a useful strategy for crop improvement. Here, we studied the regulation of the miR156 module and its interaction with SPL factors to understand the developmental transition of various plant species. Furthermore, this review provides a strong background for plant biotechnology and is an important source of information for further molecular breeding to optimize farming productivity.

## 1. Introduction

MiRNA is a non-coding small RNA molecule of approximately 20 to 24 nucleotides, which inhibits the mRNA of its target gene. They play a vital regulatory role in various aspects of eukaryotic development. MiRNAs are widely studied in plant growth because most miRNA targets interact with various transcription factors that can regulate plant phase transition, DNA methylation, chromatin remodelling, plant immunity, and biotic and abiotic stress response [1]. The regulation of miR156 is mediated by the translational inhibition of plant-specific SQUAMOSA PROMOTER BIDING-LIKE (SPL) transcription factors [2]. The regulation of miRNA on developmental stage changes was firstly discovered in *Caenorhabditis elegans*. Recent studies have shown that miR156 is involved in various activities, including integration of vegetative and reproductive growth and flowering time [3,4]; embryonic mode [5]; gibberellin-mediated flowering [6]; age-dependent flowering [7]; different patterns of pistil [8]; trichome distribution [9]; lateral root formation [4]; plant structure [10,11]; biosynthesis of secondary metabolites, such as anthocyanins and sesquiterpenes [12]; and development timing [13]. The expression of miR156 is important for the development of juveline plants and slowing down of phase transition [14]. Its target SPL gene regulates phase transition and adult development, such as flowering and fruiting [15]. Generally, miR156 downregulates the post-transcriptional SPL gene expression through transcriptional cleavage or translational repression [16], but it also shows direct regulation of genes outside of the highly conserved SPL gene family [17], including a basic leucine zipper (bZIP) transcription factor in corn [18]. Transgenic plants with the overexpression (OE) of miR156 lead to a prolonged juvenile period, delayed growth, changes in biomass, and delayed flowering [19,20]. MiR156 abundance may alter the plant trophic phase transition. The level of miR156 is increased by photosynthesis reduction and decreased by exposure to exogenous sugars. A previous finding showed that miR156 load changes are mainly attributed to the sensitive MiR156 genes [21]. The genome analysis shows that the miR156 regulatory range is highly conserved during plant evolution [22]. Research on plant structure has been carried out in various species, including *Arabidopsis*, rice, and other plants. In rice, miR156 expression is regulated by SPL genes that facilitate the growth transition from juvenile to adult, budding, leaf forming, and vegetative processes [23]. In *Triticum aestivum*, upregulation of miR156 increasese susceptiblity to heat stress [24], and its overexpression enlarges plant biomass in switchgrass. Besides, overexpression of miR156 improves the degree of biosynthesis of anthocyanin in *Arabidopsis* [25], which has been reported to alleviate the susceptibility of plants to salt and drought stress [26]. The functions of miR156 in drought, salt, and cold stress were unveiled in several studies by high-throughput microarray and microRNA sequencing [27]. miR156 shows sensitivity to drought stress in root tissues of *Triticum dicocoides* [28]. This study summarizes the emerging understanding of the regulatory function of miR156 and its central role as a multi-stress response integrator, as well as gene regulatory network characterization and plant genetic modification.

## 2. Biogenesis of miRNA

**Figure 1:**
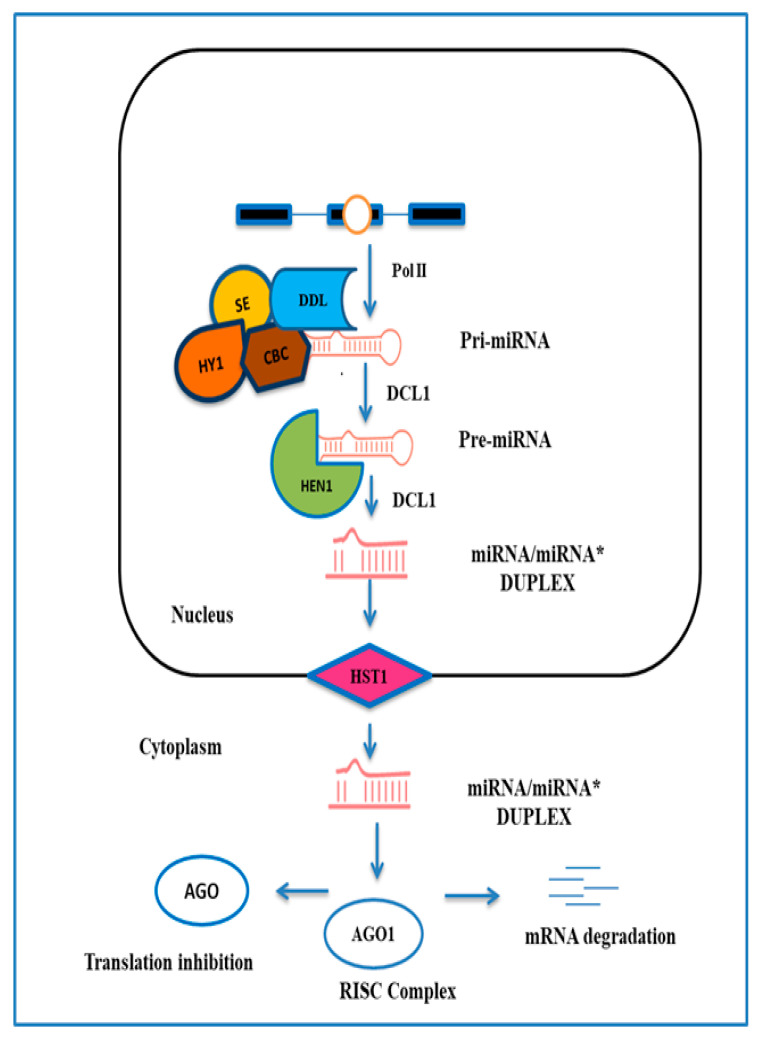
microRNA (MiRNA) biogenesis, miRNA-mediated transcriptional cleavage, and translational inhibition. A simplified model of miRNA biogenesis in plants. The miRNA gene (MIR) is transcribed into a pri-mRNA by RNA polymerase II splicing, and further processing in the nucleus involved the action of homolog of yeast longevity-1 (HYL1), dawdle (DDL), serrate (SE), and Cap-binding proteins. After methylation by the methyltransferase HUA ENHANCER1 (HEN1), the miRNA duplex is exported into the cytoplasm possibly by the HASTY (HST) and the mature miRNA strand is incorporated into an argonaute (AGO) protein.

The RNA silencing mechanism, known as co-suppression or post-transcription gene silencing, was found in plants in the early 1990s. By the end of the decade, the predictions from multiple eukaryotic models revealed the role of small RNAs in gene silencing and collectively found that RNA silencing is an evolutionary mechanism [29]. A decade after the detection of RNA silencing, distinct groups of small endogenous RNAs were defined based on their numerous sources, which are comprised of small interference RNAs (siRNAs) and miRNAs [30]. MiRNAs were first observed in *Caenorhabditis elegans*, and regulate post-transcription gene expression through small non-coding RNA (ncRNA) molecules and influence other ncRNAs, such as small interfering RNA (siRNAs) [31] and large LncRNAs [32]. miRNA targets are interpreted by miRNA response element (MRE), which are unusually complementary to the miRNA sequence in plants, and might lead to cleavage due to the high degree of complementarity but may also track genes without cleavage by physically slowing down interpretation. Like other miRNAs of the plant, miR156 is transcribed by RNA polymerase II and ensured by the mRNA cap-binding complex [33]. The precursor is processed into miRNA/miRNA* duplexes through DICER-LIKE 1 (DCL1) protein, nuclear cap-binding complex (CBC), dawdle (DDL), and serrated (SE). This duplex is then transported into the cytoplasm [34]. Then, the pre-miRNA is processed with the aid of HUA ENHANCER 1 (HEN1) protein into a mature double-stranded miRNA/miRNA * complex that is 2’-methylated at the 3’end [35]. The HASTY protein, an exportin 5 homologue, transports this small RNA duplex from the nucleus to the cytoplasm (Figure 1). The antisense miRNA * is then degraded in the cytoplasm, when the mature miRNA is embedded into an RNA-induced silencing complex (RISC). This complex is a group of ribonucleoproteins, and includes the argonaute (AGO) proteins. These AGO proteins play a role in mature miRNA/ guide strand selection and have endonuclease action against target mRNA. AGO–miRNA recognizes target mRNA within RISC, which ultimately results in either transcript cleavage or translational repression of target mRNA [36]. AGO1 has been reported to function as a slicer, whereby miRNA guides it to catalyze mRNA cleavage, but the coercive process remains uncertain. It has been observed in recent studies that the multi-step mechanism of biogenesis and degradation of miRNA plays a pivotal role in the “fine tuning” of the plant’s initial response to growth and development under environmental stress [37]. In addition, we also noticed the various components of the miR156 biogenesis pathway, such as D-body proteins, DCL1, AGO, HYL1 (homolog of yeast longevity-1), SE (serrate), DRB2 (double-stranded RNA binding protein 2), and miRNA–miRNA * duplex. Their interactome also plays a vital role in producing the ultimate response to external and internal perturbations at the plant level [38]. Therefore, miRNAs regulate transcription factors because most conserved plant miRNAs play a key role in suppressing transcription factors [39].

## 3. miR156 Targets: The Squamosa Promoter Binding Protein-Like (SBP/SPL) Family Proteins

**Figure 2:**
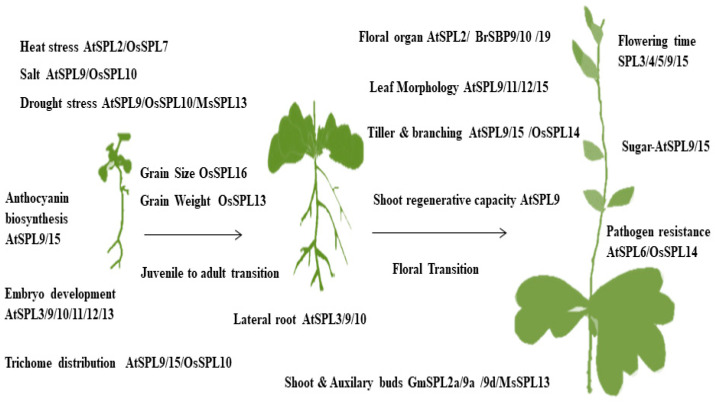
Summary of miR156/SPL-mediated plant development, including both vegetative and reproductive stages. The miR156/SPL module serves as a multifunctional gear for plant growth and development, which controls a large number of important agronomic traits.

MiR156 has agronomic relevance for plant growth and stress tolerance among a wide variety of plant miRNAs. Genomic research of MiR156 showed a highly conserved regulatory range during plant evolution [40]. SPL proteins are relatively versatile in conserved residues. Still, all of these proteins comprise a highly conserved region of 76 amino acids known as the SBP domain [41,42] consisting of two Zn-finger binding sites and a nuclear localization signal (NLS). The first identified SPL proteins were AmSBP1 and AmSBP2 of the same origin as the *Antirrhinum majus AP1* gene, which binds to the promoter of early plant flowering by the SQUAMOSA identity gene [43]. A large gene family in plants encodes SPL, for instance, there are 16 SPL genes in *Arabidopsis thaliana* [44], 19 in rice [45], and 31 in maize [46]. In rice and *Arabidopsis*, the SPL family contains miR156 MRE recital, which is typically found in the last exon behind the *SBP* domain [47]. These MREs are highly complementary to miR156, but sometimes show 1–3-nt mismatches with that of miRNA [48]. All other miR156-targeted SPL transcripts have MRE target sites in their coding regions [49]. The characterization of miR156 shows that it is an important regulatory element in various biological processes of plants, including plant growth, development, phase change, environmental stress response, and defense (Figure 2). This regulatory function of miR156 inspired the idea of applying miRNA-based genetic modification technologies to crop improvement. In plants, miR156 induces a series of phenotypic variations by regulating SPL genes. These changes include the plant height, number of branches, leaf morphology, trichome density, stem thickness, biomass yield, grain yield, and stress response [50,51].

## 4. MicroRNAs in Plant Development

**Figure 3:**
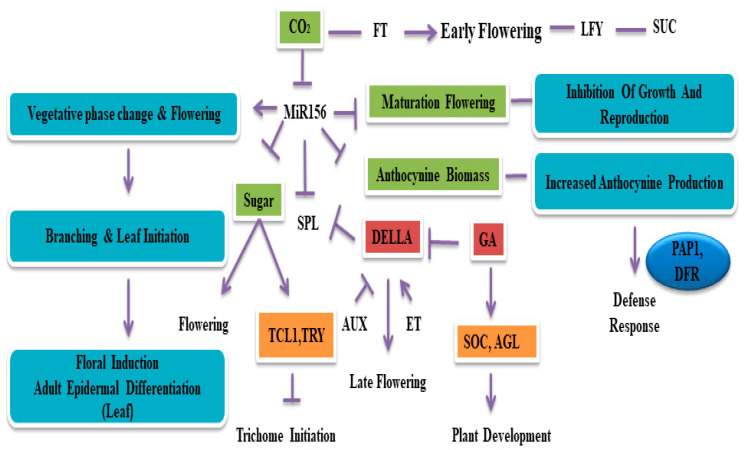
Applications of miR156/SPL gene regulation for crop improvement. miR156/SPL interaction promotes vegetative phase transition to flowering, branching/mineralization, and new leaf germination. Many endogenous and environmental cues can also accelerate phase transitions. Recent findings also indicate that plant hormones, such as auxin and ethylene, regulate the expression of miR156. The negative regulation of miR156 by sugars ensures flowering and trichome initiation. The DELLA proteins interact and repress SPL protein activities. Therefore, miR156/SPL module plays a versatile role in genetic crop improvement.

An increasing amount of evidence has shown that miR156 regulates phase transitions, root architecture, leaf and branch development, and flower development. These traits are considered essential for improving plant growth and yield. Here, we discuss each specific target trait genetic modification with a direct or indirect effect of miRNA56 (Figure 3).

### 4.1. Role of the miR156/SPL Regulatory Pathway in Phase Transition

The life cycle of a plant includes two main phase transitions. The first stage involves the transition from the juvenile to adult phase, while the second stage involves the transition from the adult to the reproductive phase. Two major microRNA species, miR156 and miR172, are responsible for controlling these phase transtions [52,53]. The relative level of miR156 was found to be higher in the seedling stage and gradually decreases with time, while the expression of miR172 was found to be lower in the early stage and steadily increases with plant growth and development [54,55]. In *Arabidopsis*, plants overexpressing miR156 showed a prolonged juvenile phase [56]. At the same time, the miR156 knockout line had a significantly shortened juvenile phase [21], indicating that miR156 is the main regulatory factor of vegetative phase transition. In the juvenile shoot, the level of miR156 is higher, resulting in lower levels of SPL. The gradual downregulation of miR156 expression during development of shoots leads to upregulation of SPL expression, thereby induceing phase transition from a juvenile to a plant [52]. The involvement of miR156 in vegetative development and flowering has been demonstrated in maize and rice [57]. The Cornrass1 mutation conferred in the maize juvenile stage is caused by constitutive expression of miR156B/C [54,57]. The correct timing of the developmental transition is critical for the survival and reproduction success of plants.

The phase transition is regulated by the expression of miR156 between the juvenile and adult nutritional stages of various plants [54,57]. Recently, hetrchronic phenotypes have been reported in maize due to overexpression of the miR156 gene [57,58]. In rice, overexpression of miR156 can result in a phenotype that is very similar to Cg, Tp1, or Tp2 mutations [45]. Higher expression of miR156 reduced the transcription of the SPL gene in *Populus canadensis* and prolonged its juvenile stage [59]. Despite the morphological diversity between species, the miR156-SPL pathway seems to be the conserved as a master switch in heteroblasty. This is also manifested in the temporal control of the distribution of trichomes in *Arabidopsis* [60], and age-dependent regulation of leaf complexity in *Cardamine hirsuta* [61] and the morphology of immature to adult leaf cells in maize [57]. These observations together with the findings revealed that miR156 and miR172 with their respective targets function in both vegetative and reproductive maturation, and imply a close connection between these two developmental transitions, although they are regulated by distinct genetic pathways.

### 4.2. Regulation of Development by Sugar Nutritional State of miR156/SPL

Glucose metabolism regulates the expression of miR156 in developing primordia. The endogenous signal that controls the vegetative phase transition may be a sugar, a key product of photosynthesis. Since then, many sugars, including glucose, sucrose, fructose, and maltose, have been repoted to promote the phase transition from a juvenile to an adult by inhibiting the expression of miR156 [21]. Methods have been developed to reduce the expression of miR156 in various processes, and the use of leaf-derived sucrose as the main determinant of annual changes in the vegetative period of the species has been proposed. In addition, a study also showed that accelerating the sugar content will prolong the germination time, promote flower induction, and enhance plant senescence [62]. However, the molecular process by which sugar inhibits miR156 is largely unclear, which requires protein synthesis and involves transcription and post-transcriptional repression. The HEXOKINASE1 (HXK1) glucose sensor catalyzes the production of glucose 6-phosphate (G6P) and also acts as a transcriptional regulator. A HXK1-null mutant gin2-1 showed a reduced level of miR156, but most of them were restored through the enzymatic inactivation process [21]. Additionally, miR156 has been proven to repress trehalose-6-phosphate (glucose used for energy storage). G6P is a substrate for trehalose phosphate synthase and repressed by miR156 following conversion to T6P with glucose and sucrose [63]. Such a mechanism will provide a reasoning for focusing on the miR156 protein synthesis of exogenous sugar suppression, which is required to increase T6P after exogenous sugar use [64]. As a result, SPL9 and SPL15 transcription rates were found to have increased dramatically as did the SPL9-GUS transcription rates. However, the expression of SPL9-GUS fusion transcripts lacking functional miR156 response factors does not affect sugar [21]. Therefore, as the juvenile plant ages, sugars produced by photosynthesis will accumulate, thereby inhibiting miR156. This leads to increased SPL expression, which then promotes the transition to the adult phase.

### 4.3. Importance of GA and DELLA Regulation in miR156/SPL

Another endogenous signal, gibberellin (GA), has gained attention for many years. It plays a variety of roles in plant growth and development, including seed germination, stem and leaf morphology, and flowering time. The role of GA has also been observed in the adjustment of phase transitions and phase traits. Among different species, it has been shown to accelerate the vegetative phase transition and flowering [65]. The mechanism of its action has recently been confirmed, in which GA acts through the DELLA protein that inhibits SPL, but GA-mediated degradation of DELLA upregulates SPL and activates flowering transition [6]. DELLAs are localized in the nucleus, and, with the help of other protein interactions, they may inhibit growth through synergistic DNA binding activity or transcriptional regulation activity, which is essential for maintaining different plant development processes [23]. The important signal transmission relationship of DELLA has been known as a negative regulator of GA [66,67]. GA is identified by a nuclear receptor called GID that binds to DELLA. GID-DELLA binds to transcription factors that promote the ubiquitination and proteolysis of DELLAs and trigger their chelation. Therefore, miR156 overexpression increased the levels of DELLA and GA-decomposing enzymes [68] but also eliminated the mutational background of miR156 non-targeting SBP, resulting in a stronger phenotype [69]. A study indicated that the regulation of SBP by GA-DELLA is mainly downstream of miR156 regulation [70]. Recent findings have also shown that phase transition may also be regulated by other hormones that may bind to GA or interact with DELLA protein, such as abscisic acid (ABA), auxin (AUX), cytokinin (CK), and jasmonic acid (JA) [71].

### 4.4. Role of the miR156/SPL Regulatory Pathway in Flowering

During developmental processes, some SPL genes targeting miR156 play a redundant role in controlling the phase transition from a juvenile to an adult, which makes the plants sensitive to photoperiod-induced flowering. Disruption of certain SPL genes prolongs the juvenile period, while premature expression of other SPL genes leads to early flowering. As mentioned earlier, the expression of miR156 gradually decreases with age, and the expression level of its target SPL also varies with age [7]. Phase transitions have shown a high level of protection in the regulation mechanism of flowering time, especially in some of specific monocots [70].

Many plant species in temperate regions, including dicots and monocots, require continuous low-temperature environmental signals (vernalization) to stimulate plant growth. The beginning of flowering indicates the transition of plant growth from vegetative growth to reproductive growth. This process is essential for successful reproduction, especially for allogeneic plants because they require synchronized flowering time with each other [72]. Overexpression of miR156 leads to prolonged juvenile and delayed flowering of many plant species, such as potato [10], alfalfa [17], tomato [73], rice [45], and corn [57]. Suppression of flowering also depends on several other transcription factors, miRNAs, and flowering genes. In *Arabidopsis*, AtSPL3/4/5 can directly activate three transcription factors, namely green leaf (LFY), fruit (FUL), and Apetala1 (AP1), that are crucial for flowering regulation, because upregulation of these regulatory factors can promote flowering [74]. AtSPL9 promotes flowering by activating miR172, which inhibits AP2 flowering repressor genes, such as EAT1 TARGET (TOE1), TOE2, TOE3 SCHLAFMUTZE (SMZ), and SCHNARCHZAPFEN [23,75]. It is proposed that these AP2-type genes can inhibit the expression of FLOWERING LOCUS T (FT), which is a flowering time inducer and can activate AP1 and FUL genes [76]. Conversely, GmmiR156b can also target SPL and negatively regulate GmSPL, which upregulates the promoters of plant-related genes in soybeans, which causes a delay in flowering time [77]. The expression of BrSBP9, BrSBP10, and BrSBP19 in flowers is more abundant than in any other tissues, which indicates that these are essential in the flowering process [78]. The overexpression of miR156 in a variety of plant species, such as *P. virgatum* [19] and *M. sativa* [79], led to drastic changes in phenotypes, such as reduced plant height, increased branching, reduced organ size, and delayed flowering. These results exposed that miR156 and its target gene SPL also play an important role in flowering.

### 4.5. Role of miR156/SPL Regulatory Pathway in Branching and Internode Growth

Branching and internode growth are important traits because they are essential for light reception. The branching of shoots entails two stages, namely the formation of axillary buds in the leaf axils and subsequent growth [80]. Various phytohormones, such as cytokinin and strigolactone, can finely regulate branching through the TB1/FC1/BRC1 pathway [81]. TEOSTINE BRANCHED 1 (TB1) and its orthologous gene FINE CLUM1 (FC1) in rice and BRANCHED 1 (BRC1) of *Arabidopsis thaliana* encodes type II TCP (TEOSINTE BRANCHED1, CYCLOIDEA, and PCF) transcription factors [23]. TB1 is a negative regulator of branching and plays an important role in branching regulation by light and other signals, such as plant hormones [81]. One of the common phenotypes of miR156 overexpression plants is enhanced branching, which gives a bushy appearance [22,82]. Like flowering control, branching is also an SPL-mediated stimulus. Overexpression of OsSPL14 (ortholog of AtSPL9) in rice promotes panicle branching [83]. In rice, miR156 affects the branching of shoots by cleaving SPL transcripts [84]. In addition, the overexpression of miR156 can cause dwarfism by inhibiting shoot apical meristems and promoting germination of axillary buds [85]. A study reported that the transcription factor ideal plant architecture1 (IPA1) encoded by OsSPL14 could promote the expression of TB1 in rice [8]. Under both long-day and short-day conditions, overexpression of miR156 induces the reduction of strigolactones contents in potatoes [10]. In soybeans, qRT-PCR results showed that the GmSPL2a, GmSPL9a, and GmSPL9d targets of GmmiR156b were significantly downregulated in the shoot tip and axillary buds [86]. Whereas, in alfalfa miR156-overexpressing plants, a decrease in internode length was also found to be accompanied by an increase in the number of nodes [82]. However, in most studies, the decrease in internode length increases the number of nodes, resulting in a reduction of plant height [17]. These findings indicate the role of the miR156-SPL module and its signal transduction in stabilizing root growth.

### 4.6. Role of the miR156/SPL Regulatory Pathway in Leaf Development

In *Arabidopsis*, 8 out of 16 SPL*s* control leaf development. Among them, AtSPL3/4/5 regulates the distribution of trichomes in the leaf. AtSPL11/12 controls the laminar shape in the vegetative phase, and AtSPL9/15 affects leaf shape [87]. Recently, it was proposed that miR156 regulates the development of trichomes of stem and floral organs by regulating the expression of its SPL target [60]. Previous studies have shown that the transcription factors R2R3 myeloblastosis(MYB) and WD40 repeat protein, basic HELIX-LOOP-HELIX (bHLH) protein, and C2H2 zinc finger protein as well as some plant hormones regulate the development of trichomes [3]. It has also been reported that AtSPL9 can affect the germination of trichomes in petals through TRICHOMELESS 1 (TCL1) [59]. A similar effect of SPL10 on trichomes was also found in *Arabidopsis* [88]. miR156 has a direct or indirect effect on all of the above-mentioned targets and regulates leaf SPL [48]. Overexpression of miR156 in plants results in smaller leaves [58,73] and an increased density [82]. In *Arabidopsis*, overexpression and mutation in a region complementary to the conserved sequence of miR156 and of SPL13 leads to excessive accumulation of miRNA-resistant transcripts and causes delayed production of leaves [89]. Therefore, SPL13, which targets miR156, is a negative regulator of leaf appearance at the cotyledon stage.

### 4.7. Role of the miR156/SPL Regulatory Pathway in Root Development and Nodulation

The miR156/SPL pathway also plays a role in root development and nodulation. Overexpression of miR156 in rice leads to the prodction of more roots [90], which suggests miR156 may play a role in root branching. A previous finding also confirmed this implication that overexpression of miR156 in *Arabidopsis* increases the number of lateral roots [4]. It has been suggested that at least one group of SPL, e.g., SPL3/9/10, is responsible for root growth, and SPL10 plays a role in inhibiting lateral root growth [4]. In addition, miR156 and SPL were found to respond to growth hormone signals, indicating their role in the development of lateral roots. It was recently discovered that the miR156/SPL module has an important role in the architecture of the root system. In the roots, it is reported that the miR156/SPL pathway affects lateral roots and adventitious roots. The reduced level of miR156 results in fewer lateral and adventitious roots [4,56]. However, the role of miR156/SPL in root development may be species specific because overexpression of MsmiR156 in alfalfa can lead to longer roots [17]. Moreover, miR156/SPL also plays a role in the nodulation of legumes. The number of nodules was found to be increased in the MsmiR156 overexpression line [17]. However, other studies have reported a reduction in nodulation reduction in miR156 overexpression plants. Contrary to the positive effect of MsmiR156, the ectopic expression of LjmiR156 (miR156 from *Lotus japonicus*) in alfalfa reduces nodules [91]. In another study conducted in soybean, it was shown that overexpression of miR156 reduces the expression of miR172, thereby increasing the number of nodules by inhibiting the AP2 transcription factor [92]. SPL6/12/13 regulates cytokinin, gibberellin, and other plant hormones and their signalling pathways, which ultimately affects root growth.

### 4.8. Role of miR156/SPL in Secondary Metabolites

In addition to plant growth and development, the role of the miR156/SPL gene regulatory network in plant secondary metabolism has also been observed. The biosynthesis of secondary metabolites is affected by many factors, including endogenous gene regulatory networks, developmental signals, and environmental stimuli [93]. In *Arabidopsis*, the relative expression of miR156 increases under heat stress [94]. In *Arabidopsis*, SPL*9* downregulates the content of anthocyanins and increases with the expression of miR156 [25]. The increase in anthocyanin content in *Arabidopsis* also leads to tolerance to abiotic stress [26], and reports on anthocyanin in rice were also found in the literature [26]. Recent studies have shown that overexpression of miR156 (miR156OE) and decreased expression of SPL13 (SPL13i) lead to an increase in the level of DIHYDROFLAVONOL 4-REDUCTASE (DFR), which encodes one of the phenylpropionic acid pathways to produce anthocyanin precursors [95]. In addition, overexpression of SPL13 changes the expression of R2R3 MYB genes, e.g., MYB53 and MYB112 [96]. Many R2R3 MYB transcription factors are involved in the phenylpropanoid pathway [25]. It has also been demonstrated in a model plant that the carotenoid content of miR156-dependent seeds is increased. For example, the overexpression of miR156 in *Brassica napus* also increases the carotenoid content of seeds [97], and *Arabidopsis* activates the marker mutation sk156, which showed an increased carotenoid content. By integrating the CaMV35S enhancer into the T-DNA insert, the sk156 mutant was developed to enhance the expression of miR156b. The increase in the carotenoid content by miR156 overexpression in plants may be due to decreased expression of CAROTENOID CLEAVAGE DIOXYGENASEs (CCDs) and decreased carotenoid catabolism, e.g., CCD8 was downregulated in SPL13-silenced alfalfa plants [96]. Recently, the role of SPL and miR156 was reported in *Arabidopsis* for spatiotemporal sesquiterpene biosynthesis [12]. It was also proved that SPL promotes the biosynthesis of sesquiterpene in old patchouli plants by activating the expression of the PATCHOULOL SYNTHASE (PatPTS) gene [12].

## 5. Functions of Plant miR156/SPL in Biotic Stress Response

At the same time, in addition to their regulation of many of the above-mentioned biological processes, the potential binding of miRNA156/SPL regualtes immune response to pathogens. Pathogens produce specific effectors, which can inhibit the first plant protection line by interrupting the pathogen associated molecular patterns (PAMP)-triggered immune (PTI) signal conversion [98]. In order to combat this pathogen strategy, plants have evolved a second line of protection, called “effector-triggered immunity” (ETI), which is enhanced by unique resistance (R) proteins [99]. The plant immune system is dependent on the growth and development of plants. At this stage, miR156/SPL plays an important role in regulating the expression of immune function genes (such as JAZ3, RPS4, ICS, and FLS2). For example, miR156-SPL9 develops resistance to *Pseudomonas syringae* during the early stage of nutrition by regulating the expression of a defense gene, i.e., FLS2. According to this view, several important genes that control plant nutrition/reproductive morphogenesis downstream of miR156/SPL can also simultaneously regulate defense genes, such as LEAFY [76] and SOC1 [100]. The development of the plant immune system is mediated by autologous transcription regulation. So far, studies focusing on a group of miR156 have revealed the role of the spatiotemporal regulatory network in the maturation of plant immune systems. Upstream, miR156 is regulated by the age-sensitive CDK8-MED12/13 mediator complex [101], and downstream, miR156 targets TF of the SPL family [2]. Because, SPL directly or indirectly regulates the expression of defense genes, the miR156-SPLs signaling module plays a central role in regulating the development of the plant immune system, thereby forming age-related immunity [102]. Moreover, miR156/SPLs modules also regulate other components of plant immunity throughout the life cycle, including the stimulation of JA signaling by stabilizing JAZ3 from UPS-mediated degradation [103,104]. Recently, artificial microRNA (amiRNA) technology has been used as a potential tool for enhancing plant resistance against various biological stresses. The phosphorylated IPA1/OsSPL14 triggers the expression of WRKY45, thereby enhancing resistance to rice blast. At the same time, unknown target genes of miR156fhl-3p may be involved in the regulation of rice blast resistance and control of various agronomic traits [105]. miR156 ensures that it plays a role in the response to various biological stresses.

## 6. Functions of Plant miR156/SPL in Abiotic Stress Response

Plant stress tolerance is a complex process that leads to protein synthesis to eliminate the destructive effects caused by stress conditions and is governed by gene regulation. The role of the transcriptional gene expression mechanism in response to plant abiotic stress has been well established. The recent development of genetically engineered plants with enhanced stress tolerance by modifying miRNA156 target nodes and promising transformation tools adapt crops to abiotic stress. Transgenic plants show enhanced tolerance to drought and salt stress by expression of the miR156-SPLs-DFR network [26]. OsSPL10 is the first OsSPL gene reported, which can regulate salt resistance in rice. Many *Os*SPL genes also confer salt resistance. In rice, overexpression of miR156 in transgenic seedlings showed a higher salt tolerance. Besides, the overexpression of miR156 in alfalfa (*Medicago sativa*) showed thicker and denser roots, resulting in increased biomass. Downregulation of SPL*13* enables the plant to survive better than non-transgenic (NT) under drought conditions [80]. In addition, compared with NT, the *Arabidopsis* mutant msc1 (more and more cells 1), resistant to miR156, showed a larger leaf size due to an increased number of cells [106]. In a study of *Brachypedes*, miR156 was upregulated under drought conditions, indicating that drought stress interferes with the normal balance between cell size and number [107]. Recently, miR156 has been shown to mediate *Arabidopsis*’s response to endure heat stress through its target SPL gene [94]. Heat stress increases the expression of miR156, thereby inhibiting the target SPL2/9/11 genes to counterbalance the negative effects of heat stress on plant growth and survival [94]. This observation indicates that the miR156/SPL regulatory module is integrated with the developmental stress response. Future research aimed at elucidating the role of miR156 in abiotic stress and its target genes will expand its effectiveness for crop improvement.

## 7. Conclusions

MiR156 and its target gene SPL transcription factor family are involved in the growth and development of phase transition, flower control, leaf shape generation, secondary metabolism, and other stress responses. So far, there are still many biological problems that need to be solved, for example: How does miR156 sense sugar signals to further regulate the phase transition? How does miR156 integrate biological stress and regulate growth? In recent years, the identification and characterization of plant miR156/SPL under biological stress has proved the importance of plant immunity. Although miR156 has been used in molecular crop breeding for several years, it still needs a clearer understanding of its gene regulatory network in order to thoroughly study the role of this molecule in improving crop quality and disease resistance. The rapid development of SPL functions achieved through specific changes in downstream target genes, or biochemical regulation of individual SPL protein functions is all the way to achieve functional variation. However, variations in specific SPL gene expression patterns confer trait variation, suggesting that this may be the main mechanism driving the genetic variation selected in plant breeding programs. Regardless of the mechanism by which SPL function regulates the nutrition and reproductive buds and the size/shape of grains, it is clear that other useful genetics were discovered and utilized variations of SPL function are now the main goal of breeders seeking to increase yields. Advances in biotechnology have provided plant breeders and researchers with many opportunities to use modern biotechnology tools to improve agronomic traits. The discovery of microRNA has added new tools to the arsenal used in plants for genetic improvement, including cereals and biomass crops.

## References

[1] Sahu P.P., Pandey G., Sharma N., Puranik S., Muthamilarasan M., Prasad M. (2013). Epigenetic mechanisms of plant stress responses and adaptation. Plant Cell Rep..

[2] Preston J.C., Hileman L.C. (2013). Functional Evolution in the Plant SQUAMOSA-PROMOTER BINDING PROTEIN-LIKE (SPL) Gene Family. Front. Plant Sci..

[3] Wang S., Chen J.-G. (2014). Regulation of cell fate determination by single-repeat R3 MYB transcription factors in Arabidopsis. Front. Plant Sci..

[4] Yu N., Niu Q.W., Ng K.H., Chua N.H. (2015). The role of miR156/SPLs modules in Arabidopsis lateral root development. Plant J..

[5] Nodine M.D., Bartel D.P. (2010). MicroRNAs prevent precocious gene expression and enable pattern formation during plant embryogenesis. Genes Dev..

[6] Yu S., Galvao V.C., Zhang Y.C., Horrer D., Zhang T.Q., Hao Y.H., Feng Y.Q., Wang S., Schmid M., Wang J.W. (2012). Gibberellin regulates the Arabidopsis floral transition through miR156-targeted SQUAMOSA promoter binding-like transcription factors. Plant Cell.

[7] Bergonzi S., Albani M.C., Ver Loren van Themaat E., Nordstrom K.J., Wang R., Schneeberger K., Moerland P.D., Coupland G. (2013). Mechanisms of age-dependent response to winter temperature in perennial flowering of *Arabis alpina*. Science.

[8] Lu Z., Yu H., Xiong G., Wang J., Jiao Y., Liu G., Jing Y., Meng X., Hu X., Qian Q. (2013). Genome-wide binding analysis of the transcription activator ideal plant architecture1 reveals a complex network regulating rice plant architecture. Plant Cell.

[9] Xue X.-Y., Zhao B., Chao L.-M., Chen D.-Y., Cui W.-R., Mao Y.-B., Wang L.-J., Chen X.-Y. (2014). Interaction between two timing MicroRNAs controls trichome distribution in Arabidopsis. PLoS Genet..

[10] Bhogale S., Mahajan A.S., Natarajan B., Rajabhoj M., Thulasiram H.V., Banerjee A.K. (2014). MicroRNA156: A potential graft-transmissible microRNA that modulates plant architecture and tuberization in *Solanum tuberosum* ssp. Andigena. Plant Physiol..

[11] Chen Z., Gao X., Zhang J. (2015). Alteration of osa-miR156e expression affects rice plant architecture and strigolactones (SLs) pathway. Plant Cell Rep..

[12] Yu Z.X., Wang L.J., Zhao B., Shan C.M., Zhang Y.H., Chen D.F., Chen X.Y. (2014). Progressive regulation of sesquiterpene biosynthesis in Arabidopsis and patchouli (*Pogostemon cablin*) by the miR156-Targeted SPL Transcription Factors. Mol. Plant.

[13] Cho S.H., Coruh C., Axtell M.J. (2012). miR156 and miR390 regulate tasiRNA accumulation and developmental timing in Physcomitrella patens. Plant Cell.

[14] Schwarz S., Grande A.V., Bujdoso N., Saedler H., Huijser P. (2008). The microRNA regulated SBP-box genes SPL9 and SPL15 control shoot maturation in Arabidopsis. Plant Mol. Biol..

[15] Wu G., Poethig R.S. (2006). Temporal regulation of shoot development in *Arabidopsis thaliana* by miR156 and its target SPL3. Development.

[16] He J., Xu M., Willmann M.R., McCormick K., Hu T., Yang L., Starker C.G., Voytas D.F., Meyers B.C., Poethig R.S. (2018). Threshold-dependent repression of SPL gene expression by miR156/miR157 controls vegetative phase change in *Arabidopsis thaliana*. PLoS Genet..

[17] Aung B., Gruber M.Y., Amyot L., Omari K., Bertrand A., Hannoufa A. (2015). MicroRNA156 as a promising tool for alfalfa improvement. Plant Biotechnol. J..

[18] Chuck G.S., Tobias C., Sun L., Kraemer F., Li C., Dibble D., Arora R., Bragg J.N., Vogel J.P., Singh S. (2011). Overexpression of the maize *Corngrass1* microRNA prevents flowering, improves digestibility, and increases starch content of switchgrass. Proc. Natl. Acad. Sci. USA.

[19] Fu C., Sunkar R., Zhou C., Shen H., Zhang J.Y., Matts J., Wolf J., Mann D.G., Stewart C.N., Tang Y. (2012). Overexpression of miR156 in switchgrass (*Panicum virgatum* L.) results in various morphological alterations and leads to improved biomass production. Plant Biotechnol. J..

[20] Shikata M., Yamaguchi H., Sasaki K., Ohtsubo N. (2012). Overexpression of Arabidopsis miR157b induces bushy architecture and delayed phase transition in *Torenia fournieri*. Planta.

[21] Yang L., Xu M., Koo Y., He J., Poethig R.S. (2013). Sugar promotes vegetative phase change in *Arabidopsis thaliana* by repressing the expression of MIR156A and MIR156C. Elife.

[22] Aung B., Gruber M.Y., Hannoufa A. (2015). The MicroRNA156 system: A tool in plant biotechnology. Biocatal. Agric. Biotechnol..

[23] Wang H., Wang H. (2015). The miR156/SPL Module, a regulatory hub and versatile toolbox, gears up crops for enhanced agronomic traits. Mol. Plant.

[24] Xin M., Wang Y., Yao Y., Xie C., Peng H., Ni Z., Sun Q. (2010). Diverse set of microRNAs are responsive to powdery mildew infection and heat stress in wheat (*Triticum aestivum* L.). BMC Plant Biol..

[25] Gou J.-Y., Felippes F.F., Liu C.-J., Weigel D., Wang J.-W. (2011). Negative regulation of anthocyanin biosynthesis in Arabidopsis by a miR156-targeted SPL transcription factor. Plant Cell.

[26] Cui L.G., Shan J.X., Shi M., Gao J.P., Lin H.X. (2014). The miR156-SPL9-DFR pathway coordinates the relationship between development and abiotic stress tolerance in plants. Plant J..

[27] Lee H., Yoo S.J., Lee J.H., Kim W., Yoo S.K., Fitzgerald H., Carrington J.C., Ahn J.H. (2010). Genetic framework for flowering-time regulation by ambient temperature-responsive miRNAs in Arabidopsis. Nucleic Acids Res..

[28] Kantar M., Lucas S., Budak H. (2010). miRNA expression patterns of *Triticum dicoccoides* in response to shock drought stress. Planta.

[29] Cogoni C., Macino G. (2000). Post-transcriptional gene silencing across kingdoms. Curr. Opin. Genet. Dev..

[30] Chapman E.J., Carrington J.C. (2007). Specialization and evolution of endogenous small RNA pathways. Nat. Rev. Genet..

[31] Manavella P.A., Koenig D., Weigel D. (2012). Plant secondary siRNA production determined by microRNA-duplex structure. Proc. Natl. Acad. Sci. USA.

[32] Zhang J., Wei L., Jiang J., Mason A.S., Li H., Cui C., Chai L., Zheng B., Zhu Y., Xia Q. (2018). Genome-wide identification, putative functionality and interactions between lncRNAs and miRNAs in *Brassica* species. Sci. Rep..

[33] Gregory B.D., O’Malley R.C., Lister R., Urich M.A., Tonti-Filippini J., Chen H., Millar A.H., Ecker J.R. (2008). A link between RNA metabolism and silencing affecting Arabidopsis development. Dev. Cell.

[34] Rogers K., Chen X. (2013). Biogenesis, turnover, and mode of action of plant microRNAs. Plant Cell.

[35] Abe M., Yoshikawa T., Nosaka M., Sakakibara H., Sato Y., Nagato Y., Itoh J. (2010). WAVY LEAF1, an ortholog of Arabidopsis HEN1, regulates shoot development by maintaining MicroRNA and trans-acting small interfering RNA accumulation in rice. Plant Physiol..

[36] Wu L., Zhou H., Zhang Q., Zhang J., Ni F., Liu C., Qi Y. (2010). DNA methylation mediated by a microRNA pathway. Mol. Cell.

[37] Millar A.A. (2020). The Function of miRNAs in Plants. Plants.

[38] Choi S.W., Ryu M.Y., Viczian A., Jung H.J., Kim G.M., Arce A.L., Achkar N.P., Manavella P., Dolde U., Wenkel S. (2020). Light Triggers the miRNA-biogenetic inconsistency for de-etiolated seedling survivability in *Arabidopsis thaliana*. Mol. Plant.

[39] Samad A.F.A., Sajad M., Nazaruddin N., Fauzi I.A., Murad A.M.A., Zainal Z., Ismail I. (2017). MicroRNA and transcription factor: Key players in plant regulatory network. Front. Plant Sci..

[40] Zhang L., Hu Y.-b., Wang H.-S., Shengjun F., Zhang Y.-T. (2015). Involvement of miR156 in the regulation of vegetative phase change in plants. J. Am. Soc. Hortic. Sci..

[41] Birkenbihl R.P., Jach G., Saedler H., Huijser P. (2005). Functional dissection of the plant-specific SBP-domain: Overlap of the DNA-binding and nuclear localization domains. J. Mol. Biol..

[42] Yamasaki K., Kigawa T., Inoue M., Watanabe S., Tateno M., Seki M., Shinozaki K., Yokoyama S. (2008). Structures and evolutionary origins of plant-specific transcription factor DNA-binding domains. Plant Physiol. Biochem..

[43] Klein J., Saedler H., Huijser P. (1996). A new family of DNA binding proteins includes putative transcriptional regulators of the *Antirrhinum majus* floral meristem identity gene SQUAMOSA. Mol. Gen. Genet..

[44] Cardon G., Hohmann S., Klein J., Nettesheim K., Saedler H., Huijser P. (1999). Molecular characterisation of the Arabidopsis SBP-box genes. Gene.

[45] Xie K., Wu C., Xiong L. (2006). Genomic organization, differential expression, and interaction of SQUAMOSA promoter-binding-like transcription factors and microRNA156 in rice. Plant Physiol.

[46] Mao H.-D., Yu L.-J., Li Z.-J., Yan Y., Han R., Liu H., Ma M. (2016). Genome-wide analysis of the SPL family transcription factors and their responses to abiotic stresses in maize. Plant Gene.

[47] Yang Z., Wang X., Gu S., Hu Z., Xu H., Xu C. (2008). Comparative study of SBP-box gene family in Arabidopsis and rice. Gene.

[48] Rhoades M.W., Reinhart B.J., Lim L.P., Burge C.B., Bartel B., Bartel D.P. (2002). Prediction of plant microRNA targets. Cell.

[49] Gandikota M., Birkenbihl R.P., Hohmann S., Cardon G.H., Saedler H., Huijser P. (2007). The miRNA156/157 recognition element in the 3’ UTR of the Arabidopsis SBP box gene SPL3 prevents early flowering by translational inhibition in seedlings. Plant J..

[50] Zhou M., Luo H. (2013). MicroRNA-mediated gene regulation: Potential applications for plant genetic engineering. Plant Mol. Biol..

[51] Padmanabhan C., Zhang X., Jin H. (2009). Host small RNAs are big contributors to plant innate immunity. Curr. Opin. Plant Biol..

[52] Wu G., Park M.Y., Conway S.R., Wang J.W., Weigel D., Poethig R.S. (2009). The sequential action of miR156 and miR172 regulates developmental timing in Arabidopsis. Cell.

[53] Huijser P., Schmid M. (2011). The control of developmental phase transitions in plants. Development.

[54] Fahlgren N., Howell M.D., Kasschau K.D., Chapman E.J., Sullivan C.M., Cumbie J.S., Givan S.A., Law T.F., Grant S.R., Dangl J.L. (2007). High-throughput sequencing of Arabidopsis microRNAs: Evidence for frequent birth and death of MIRNA genes. PLoS ONE.

[55] Wang Y., Li P., Cao X., Wang X., Zhang A., Li X. (2009). Identification and expression analysis of miRNAs from nitrogen-fixing soybean nodules. Biochem. Biophys. Res. Commun..

[56] Xu M., Hu T., Zhao J., Park M.-Y., Earley K.W., Wu G., Yang L., Poethig R.S. (2016). Developmental functions of miR156-regulated SQUAMOSA PROMOTER BINDING PROTEIN-LIKE (SPL) genes in *Arabidopsis thaliana*. PLoS Genet..

[57] Chuck G., Cigan A.M., Saeteurn K., Hake S. (2007). The heterochronic maize mutant Corngrass1 results from overexpression of a tandem microRNA. Nat. Genet..

[58] Poethig R.S. (2009). Small RNAs and developmental timing in plants. Curr. Opin. Genet. Dev..

[59] Wang J.-W., Park M.Y., Wang L.-J., Koo Y., Chen X.-Y., Weigel D., Poethig R.S. (2011). MiRNA control of vegetative phase change in trees. PLOS Genet..

[60] Yu N., Cai W.-J., Wang S., Shan C.-M., Wang L.-J., Chen X.-Y. (2010). Temporal control of trichome distribution by MicroRNA156-targeted SPL genes in *Arabidopsis thaliana*. Plant Cell.

[61] Rubio-Somoza I., Zhou C.-M., Confraria A., Martinho C., von Born P., Baena-Gonzalez E., Wang J.-W., Weigel D. (2014). Temporal control of leaf complexity by miRNA-regulated licensing of protein complexes. Curr. Biol..

[62] Gibson S.I. (2005). Control of plant development and gene expression by sugar signaling. Curr. Opin. Plant Biol..

[63] Wahl V., Ponnu J., Schlereth A., Arrivault S., Langenecker T., Franke A., Feil R., Lunn J.E., Stitt M., Schmid M. (2013). Regulation of flowering by trehalose-6-phosphate signaling in *Arabidopsis thaliana*. Science.

[64] Yadav U.P., Ivakov A., Feil R., Duan G.Y., Walther D., Giavalisco P., Piques M., Carillo P., Hubberten H.-M., Stitt M. (2014). The sucrose-trehalose 6-phosphate (Tre6P) nexus: Specificity and mechanisms of sucrose signalling by Tre6P. J. Exp. Bot..

[65] Mimura M., Nagato Y., Itoh J. (2012). Rice PLASTOCHRON genes regulate leaf maturation downstream of the gibberellin signal transduction pathway. Planta.

[66] Hyun Y., Richter R., Vincent C., Martinez-Gallegos R., Porri A., Coupland G. (2016). Multi-layered regulation of SPL15 and cooperation with SOC1 integrate endogenous flowering pathways at the Arabidopsis shoot meristem. Dev. Cell.

[67] Zentella R., Zhang Z.L., Park M., Thomas S.G., Endo A., Murase K., Fleet C.M., Jikumaru Y., Nambara E., Kamiya Y. (2007). Global analysis of della direct targets in early gibberellin signaling in Arabidopsis. Plant Cell.

[68] Sun T.-P. (2011). The molecular mechanism and evolution of the GA-GID1-DELLA signaling module in plants. Curr. Biol. CB.

[69] Xing S., Salinas M., Höhmann S., Berndtgen R., Huijser P. (2010). miR156-targeted and nontargeted SBP-Box transcription factors act in concert to secure male fertility in Arabidopsis. Plant Cell.

[70] Tanaka N. (2012). Gibberellin is not a regulator of miR156 in rice juvenile-adult phase change. Rice (N. Y.).

[71] Hibara K.-I., Isono M., Mimura M., Sentoku N., Kojima M., Sakakibara H., Kitomi Y., Yoshikawa T., Itoh J.-I., Nagato Y. (2016). Jasmonate regulates juvenile-to-adult phase transition in rice. Development.

[72] Spanudakis E., Jackson S. (2014). The role of microRNAs in the control of flowering time. J. Exp. Bot..

[73] Zhang X., Zou Z., Zhang J., Zhang Y., Han Q., Hu T., Xu X., Liu H., Li H., Ye Z. (2011). Over-expression of sly-miR156a in tomato results in multiple vegetative and reproductive trait alterations and partial phenocopy of the sft mutant. FEBS Lett..

[74] Yamaguchi A., Wu M.F., Yang L., Wu G., Poethig R.S., Wagner D. (2009). The microRNA-regulated SBP-Box transcription factor SPL3 is a direct upstream activator of LEAFY, FRUITFULL, and APETALA1. Dev. Cell.

[75] Yamaguchi A., Abe M. (2012). Regulation of reproductive development by non-coding RNA in Arabidopsis: To flower or not to flower. J. Plant Res..

[76] Mathieu J., Warthmann N., Kuttner F., Schmid M. (2007). Export of FT protein from phloem companion cells is sufficient for floral induction in Arabidopsis. Curr. Biol..

[77] Cao D., Li Y., Wang J., Nan H., Wang Y., Lu S., Jiang Q., Li X., Shi D., Fang C. (2015). GmmiR156b overexpression delays flowering time in soybean. Plant Mol. Biol..

[78] Tan H.W., Song X.M., Duan W.K., Wang Y., Hou X.L. (2015). Genome-wide analysis of the SBP-box gene family in Chinese cabbage (*Brassica rapa* subsp. *pekinensis*). Genome.

[79] Arshad M., Gruber M.Y., Wall K., Hannoufa A. (2017). An Insight into microRNA156 role in salinity stress responses of Alfalfa. Front. Plant Sci..

[80] Beveridge C.A., Kyozuka J. (2010). New genes in the strigolactone-related shoot branching pathway. Curr. Opin. Plant Biol..

[81] Kebrom T.H., Spielmeyer W., Finnegan E.J. (2013). Grasses provide new insights into regulation of shoot branching. Trends Plant Sci..

[82] Gao R., Austin R.S., Amyot L., Hannoufa A. (2016). Comparative transcriptome investigation of global gene expression changes caused by miR156 overexpression in *Medicago sativa*. BMC Genom..

[83] Miura K., Ikeda M., Matsubara A., Song X.J., Ito M., Asano K., Matsuoka M., Kitano H., Ashikari M. (2010). OsSPL14 promotes panicle branching and higher grain productivity in rice. Nat. Genet..

[84] Liu J., Cheng X., Liu P., Sun J. (2017). miR156-Targeted SBP-box transcription factors interact with DWARF53 to regulate TEOSINTE BRANCHED1 and BARREN STALK1 expression in bread wheat. Plant Physiol..

[85] Wang L., Sun S., Jin J., Fu D., Yang X., Weng X., Xu C., Li X., Xiao J., Zhang Q. (2015). Coordinated regulation of vegetative and reproductive branching in rice. Proc. Natl. Acad. Sci. USA.

[86] Sun Z., Su C., Yun J., Jiang Q., Wang L., Wang Y., Cao D., Zhao F., Zhao Q., Zhang M. (2019). Genetic improvement of the shoot architecture and yield in soya bean plants via the manipulation of GmmiR156b. Plant Biotechnol. J..

[87] Chen X., Zhang Z., Liu D., Zhang K., Li A., Mao L. (2010). SQUAMOSA Promoter-binding protein-like transcription factors: Star players for plant growth and development. J. Integr. Plant Biol..

[88] Shikata M., Koyama T., Mitsuda N., Ohme-Takagi M. (2009). Arabidopsis SBP-box genes SPL10, SPL11 and SPL2 control morphological change in association with shoot maturation in the reproductive phase. Plant Cell Physiol..

[89] Martin R.C., Asahina M., Liu P.-P., Kristof J.R., Coppersmith J.L., Pluskota W.E., Bassel G.W., Goloviznina N.A., Nguyen T.T., Martínez-Andújar C. (2010). The microRNA156 and microRNA172 gene regulation cascades at post-germinative stages in Arabidopsis. Seed Sci. Res..

[90] Xie K., Shen J., Hou X., Yao J., Li X., Xiao J., Xiong L. (2012). Gradual increase of miR156 regulates temporal expression changes of numerous genes during leaf development in rice. Plant Physiol..

[91] Aung B., Gruber M.Y., Amyot L.M., Omari K., Bertrand A., Hannoufa A. (2015). Ectopic expression of LjmiR156 delays flowering, enhances shoot branching, and improves forage quality in alfalfa. Plant Biotechnol. Rep..

[92] Yan Z., Hossain M.S., Wang J., Valdes-Lopez O., Liang Y., Libault M., Qiu L., Stacey G. (2013). miR172 regulates soybean nodulation. Mol. Plant Microbe Interact..

[93] Lv Q., Cheng R., Shi T. (2014). Regulatory network rewiring for secondary metabolism in *Arabidopsis thaliana* under various conditions. BMC Plant Biol..

[94] Stief A., Altmann S., Hoffmann K., Pant B., Scheible W., Bäurle I. (2014). Arabidopsis miR156 regulates tolerance to recurring environmental stress through SPL transcription factors. Plant Cell.

[95] Davies K.M., Schwinn K.E., Deroles S.C., Manson D.G., Lewis D.H., Bloor S.J., Bradley J.M. (2003). Enhancing anthocyanin production by altering competition for substrate between flavonol synthase and dihydroflavonol 4-reductase. Euphytica.

[96] Gao R., Gruber M.Y., Amyot L., Hannoufa A. (2018). SPL13 regulates shoot branching and flowering time in *Medicago sativa*. Plant Mol. Biol..

[97] Wei S., Yu B., Gruber M.Y., Khachatourians G.G., Hegedus D.D., Hannoufa A. (2010). Enhanced seed carotenoid levels and branching in transgenic *Brassica napus* expressing the Arabidopsis miR156b Gene. J. Agric. Food Chem..

[98] Monaghan J., Zipfel C. (2012). Plant pattern recognition receptor complexes at the plasma membrane. Curr. Opin. Plant Biol..

[99] Baldrich P., San Segundo B. (2016). MicroRNAs in rice innate immunity. Rice (N. Y.).

[100] Lee J., Oh M., Park H., Lee I. (2008). SOC1 translocated to the nucleus by interaction with AGL24 directly regulates leafy. Plant J..

[101] Gillmor C.S., Silva-Ortega C.O., Willmann M.R., Buendia-Monreal M., Poethig R.S. (2014). The Arabidopsis mediator CDK8 module genes CCT (MED12) and GCT (MED13) are global regulators of developmental phase transitions. Development.

[102] Zheng J., Ma Y., Zhang M., Lyu M., Yuan Y., Wu B. (2019). Expression Pattern of FT/TFL1 and miR156-targeted SPL genes associated with developmental stages in *Dendrobium catenatum*. Int. J. Mol. Sci..

[103] Gaquerel E., Stitz M. (2017). Insect resistance: An emerging molecular framework linking plant age and JA signaling. Mol. Plant.

[104] Mao Y.-B., Liu Y.-Q., Chen D.-Y., Chen F.-Y., Fang X., Hong G.-J., Wang L.-J., Wang J.-W., Chen X.-Y. (2017). Jasmonate response decay and defense metabolite accumulation contributes to age-regulated dynamics of plant insect resistance. Nat. Commun..

[105] Wang J., Zhou L., Shi H., Chern M., Yu H., Yi H., He M., Yin J., Zhu X., Li Y. (2018). A single transcription factor promotes both yield and immunity in rice. Science.

[106] Usami T., Horiguchi G., Yano S., Tsukaya H. (2009). The more and smaller cells mutants of Arabidopsis thaliana identify novel roles for SQUAMOSA PROMOTER BINDING PROTEIN-LIKE genes in the control of heteroblasty. Development.

[107] Bertolini E., Verelst W., Horner D.S., Gianfranceschi L., Piccolo V., Inze D., Pe M.E., Mica E. (2013). Addressing the role of microRNAs in reprogramming leaf growth during drought stress in *Brachypodium Distachyon*. Mol. Plant.

